# Elevated circulating high-sensitivity cardiac troponin t and cardiac remodeling in obesity

**DOI:** 10.1186/s12872-021-02445-0

**Published:** 2021-12-28

**Authors:** Jiaojiao Huang, Ming Liu, Enyong Su, Peng Yu, Hong Jiang, Ji Zhao, Junbo Ge

**Affiliations:** 1 Department of General Practice, Zhongshan Hospital, Shanghai Medical College of Fudan University, Shanghai, China; 2 Department of Health Management Center, Zhongshan Hospital, Shanghai Medical College of Fudan University, Shanghai, China; 3 Shanghai Institute of Cardiovascular Diseases, Shanghai Institute of Clinical Bioinformatics, Zhongshan Hospital, Shanghai Medical College of Fudan University, Shanghai, China; 4 Department of Endocrinology and Metabolism, Zhongshan Hospital, Shanghai Medical College of Fudan University, Shanghai, China

**Keywords:** Obesity, High-sensitivity cardiac troponin T, Myocardial injury, Coronary heart disease

## Abstract

**Background:**

It is well established that body mass index (BMI) and troponins are independently associated. However, whether the obesity could cause myocardial injury independent of coronary heart disease (CHD) remains unclear. This study focuses on the relationship between BMI and troponins, and whether this relationship is being attenuated when CHD is accounted for.

**Methods:**

In populations without acute ischemic events, 383 patients with coronary artery stenosis less than 75% were included, that is, people who have not yet reached the indications for coronary intervention, and of them 70 patients being obese according to BMI ≥ 28 kg/m^2^. Continuous variables were represented as mean ± SD or median(inter quartile range[IQR]). Chi-square test was adopted for categorical data. Correlations between variables were evaluated by Spearman analysis, multiple regression or logistic regression.

**Results:**

The circulating hs-cTnT level was higher in the obese group [8(6,11) ng/L vs. 6(4,9) ng/L; *p* < 0.001). In subgroup analysis based on the presence or absence of coronary heart disease(CHD), the adjusted β(95%CI) for circulating hs-cTnT exhibited a proportional relationship with BMI when the non-obesity were defined as the reference[β; 2.22(95%CI, 0.73 to 3.71) in non-CHD, 5.58(95%CI, 0.70 to 10.46) in CHD*, p* < *0.05*]. Additionally, the degree of coronary stenosis has shown a positive correlation with circulating hs-cTnT (rho = 0.1162; *p* < 0.05).

**Conclusion:**

When CHD is taken into account, obesity is independently associated to the elevation of circulating hs-cTnT, a biomarker of myocardial injury, potentially indicating the impact of obesity on non-ischemic subclinical myocardial injury.

## Introduction

Obese men and obese women respectively account for 16.3% and 12.4% globally, and maintain a steady growth trend. Admittedly, obesity is closely associated with the development and occurrence of cardiovascular disease (CVD) [1, 2] including: cardiac artery disease (CAD) and non-ischemic heart disease. Meanwhile, obesity may often accompany with myocardial injury, which indicates a worse prognosis for CVD.

Myocardial injury may be led by two factors. On the one hand, the CAD including stable angina pectoris and acute coronary syndrome (ACS) could cause damage to myocardium due to myocardial ischemia [3]. On the other hand, myocardial injury would inevitably come with the non-ischemic heart disease such as cardiac remodeling or dysfunction diseases. Current studies [4, 5] show that obesity is independently associated with cardiac remodeling, with left ventricular structural abnormalities-related indicators having a dose–response relationship with body mass index (BMI). Meanwhile, obesity is a culprit behind the occurrence of heart failure (HF), manifested by diastolic HF and subsequent systolic HF [6]. However, to our best knowledge, few studies directly explore the relationship between obesity and subclinical myocardial injury, taking coronary artery diseases into account. A study based on National Health and Nutrition Examination Survey (NHASNES) III suggested that obesity is positively associated with subclinical myocardial injury [7]. But what is more noteworthy is that there are virtually no studies excluding the effects of coronary vascular lesions on myocardial injury through the gold standard coronary angiography to prove the correlation between obesity and myocardial injury. Additionally, circulating cardiac Troponin T (cTnT) especially for circulating high-sensitivity cardiac Troponin T (hs-cTnT), an extensively acknowledged biomarker for myocardial injury in today’s clinical settings, routinely serves as a diagnostic tool for ACS when it exceeds the 99th percentile of the upper limit of the refence value at least once [8]. Meanwhile, its slight elevation might suggest the minor damage on cardiac muscle. And with time, the elevation in circulating hs-cTnT would have a predictive effect on the occurrence of events and a higher mortality risk [9, 10].

The aim of this cross-sectional study was to find whether there existed an independent correlation between obesity and slightly elevated circulating hs-cTnT under the premise of excluding the influence of CHD, which may be the evidence on non-ischemic subclinical myocardial injury caused by obesity.

## Methods

### Study population

Patients who underwent coronary angiography due to long-term chronic chest discomfort at Zhongshan Hospital ranging from December 1st, 2020 to May 30th, 2021 at Fudan University were screened in the study. Patients with coronary artery stenosis less than 75%, that is, non-coronary heart disease (non-CHD) patients (coronary artery stenosis less than 50%) and coronary heart disease patients who did not meet the indications for intervention (coronary artery stenosis less than 75% but more than or equal to 50%) were consecutively enrolled. Exclusion criteria were as follows [11]: incomplete clinical data, acute myocardial ischemic event (ACS on admission), a history of cardiovascular events (including stroke, ACS or HF), structural cardiomyopathy, atrial fibrillation or flutter, atrioventricular block and tachycardia, implanted pacemakers, sepsis, bronchial or lung disease, hyperthyroidism and end-stage renal disease. The calculation of sample sizes was based on previous literature [12], of which the circulating hs-cTnT stood at 2.3 (1.5, 3.7)pg/ml (equal to ng/L), in the non-metabolic syndrome group and 3.4 (2.2, 4.6)pg/ml in the metabolic syndrome group, and the tool [13] https://www.math.hkbu.edu.hk/~tongt/papers/median2mean.html was used in order to transform the data into mean ± SD, respectively being 2.51 ± 1.63 pg/ml and 3.40 ± 1.80 pg/ml after the transformation. Then we input the syntax to the STATA, namely *sampsi 2.51 3.4, sd1 (1.63) sd2 (1.8) power (0.80) alpha (0.05) ratio (4)*. The results were n_1_ = 35 and n_2_ = 140. The number of enrolled patients was twice as much as the minimum requirement.

The study was approved by the Ethical Committee of Zhongshan Hospital, Fudan University, China and all participants had signed informed consent, which was in line with the principles declared in the 1975 Declaration of Helsinki.

### Data collection

We retrospectively collected data on demographic characteristics, symptoms and signs, previous combined diseases, coronary angiography and Doppler ultrasound results. All blood samples were obtained on the first morning of hospitalization after an overnight fast for laboratory measurements. Laboratory data of peripheral venous blood including blood cell counts, inflammation indicators, cardiac function, hepatic function, renal function, thyroid function, lipids and sugar levels are recorded. All results were checked by another researcher.

The blood pressure (BP) of patients was measured repeatedly three times at an interval of two minutes in the morning by Omron electric sphygmomanometer (U30; Omron Dalian Co., Ltd; Dalian; China) and we record the average value. Hypertension was defined as those with mean systolic blood pressure (SBP) ≥ 140 mmHg and/or mean diastolic blood pressure (DBP) ≥ 90 mmHg or taking antihypertensive drugs. Diabetes mellitus was characterized as hemoglobin A1c (HbA1c) ≥ 6.5%, fasting blood glucose (FBG) ≥ 7.0 mmol/L, 2-h plasma glucose ≥ 11.1 mmol/L, a random plasma glucose ≥ 11.1 mmol/L or receiving hypoglycemic treatments. Individuals with high-density lipoprotein cholesterol (HDL-C) ≤ 40 mg/dL, low-density lipoprotein cholesterol (LDL-C) ≥ 140 mg/dL, triglycerides ≥ 150 mg/dL or taking lipid-lowering medications were listed as hyperlipidemia. Body mass index (BMI) refers to the weight in kilograms divided by the square of the height in meters. Obesity was defined as BMI ≥ 28 kg/m^2^ [14].

### Biochemical measurements

The level of circulating hs-cTnT is obtained using a high-sensitivity and high-precision cardiac troponin assay-Troponin T hs STAT (Roche Diagnostics GmbH Shanghai Co., Ltd, Shanghai, China) by electrochemiluminescence in the Cobas e601(Roche Diagnostics GmbH Shanghai Co., Ltd, Shanghai, China). Circulating hs-cTnT’s normal reference value range stands at 0–14 ng/L, with its lower limit of detection being 3 ng/L [9].

### Echocardiographic measurement

Echocardiograms were measured using the EPIQ 7C Ultrasound System (Phillips Medical Systems, Bothell, Washington) with a 1.0- to 5.0- MHz transducer based on the recommendation of the American Society of Echocardiography [15]. All patients were recommended to lie in a left lateral recumbent position. In the parasternal long and short axes and the apical 4- and 2- chamber long-axis perspective, two-dimensional and Doppler images were recorded. From the parasternal perspective, M-mode echocardiograms of the left ventricle (LV) were obtained. All records averaged at least 3 cardiac cycles and were digitally stored for subsequent statistical analysis. Left ventricular end-systolic diameter (LVESD), left ventricular end-diastolic diameter (LVEDD), posterior wall thickness (PWT) and interventricular septal thickness (IVST) were measured using M-mode or 2-dimensional echocardiography from the parasternal long axis view. Left atrial dimension (LAD) were measured in 3 orthogonal planes: the parasternal long, lateral and super-inferior axes. Left ventricular ejection (LVEF) was calculated from LV end-systolic and end-diastolic volumes (LVESV and LVEDV) estimated from the apical 4- and 2- chambers perspectives by Simpson biplane method. The deceleration time (DT) of early diastolic velocity were recorded as indicators of LV end-diastolic pressure.

### Statistical analysis

All continuous variables were tested for normality using Kolmogorov–Smirnov test. If being normal distribution, the data were represented as mean ± SD and compared by the Student’s test. Otherwise, the data were reported as median (inter quartile range [IQR]) and the Mann–Whitney U-test. Chi-square test is adopted for categorical data. Correlation between obesity and circulating hs-cTnT was evaluated by multiple linear regression, with hs-cTnT serving as the dependent variable. For association between parameters in echocardiography and circulating hs-cTnT or BMI, spearman correlation analysis was practiced. *P* value < 0.05 was considered statistically significant. Stata 12 for windows (Stata-Corp, College Station, TX, USA) was used during the statistical analysis process.

## Results

### Demographical and clinical characteristics

A total of 383 patients with coronary artery stenosis less than 75% were included in the study. Of them, 313 (81.7%) were non-obese and the remaining 70 (19.3%) individuals were obese. The mean age was 61.6 ± 0.5 years and male patients stood at 218 (56.9%). Demographical and clinical characteristics of the participants were shown in Table 1. The obesity group tended to have more unfavorable metabolic profiles, namely, the obese had higher SBP, DBP and HbA1c than their counterparts. However, the obese showed lower HDL-c compared with the non-obese. Notably, circulating hs-cTnT were apparently higher in obese group than non-obese group. Besides, alanine transaminase (ALT) and C-reactive protein (CRP) were higher in obesity group. Other factors such as D-dimer, isoenzyme of creatine kinase (CK-MB), aspartate transaminase (AST), glomerular filtration rate (GFR), creatinine, total cholesterol (TC), total triglycerides (TG), LDL-c and thyrotropin-releasing hormone (TSH) did not show statistical differences.

**Table 1:** Clinical characteristic of 383 patients

	BMI		t/z/ value	*P* value
	Non-obesity (n = 313)	Obesity (n = 70)		
Age (years old, ${\overline{\text{x}}} \pm {\text{s}}$)	61.91 ± 9.33	59.80 ± 9.36	1.71	0.09
Male (number, %)	177 (56.55)	41 (58.57)	0.10^a^	0.76
Height (cm, ${\overline{\text{x}}} \pm {\text{s}}$)	165.45 ± 7.79	164.67 ± 9.39	0.64	0.52
Weight (kg, ${\overline{\text{x}}} \pm {\text{s}}$)	66.52 ± 9.68	82.36 ± 9.80	− 12.34	< 0.001*
Systolic BP (mmHg, ${\overline{\text{x}}} \pm {\text{s}}$)	129.89 ± 18.33	135.46 ± 18.25	− 2.3	0.02^*^
Diastolic BP (mmHg, ${\overline{\text{x}}} \pm {\text{s}}$)	79.02 ± 10.86	82.33 ± 9.23	− 2.36	0.02*
DM (number, %)	56 (17.89)	14 (20.00)	0.17^a^	0.68
HBP (number, %)	29 (15.68)	41 (20.71)	1.62^a^	0.2
Coronary stenosis[%,M(Q_1_,Q_3_)]	30 (0,50)	30 (0, 40)	1.52^b^	0.13
Hyperlipidemia (number, %)	31 (9.90)	6 (8.57)	0.12^a^	0.73
hs-cTnT(ng/L, ${\overline{\text{x}}} \pm {\text{s}}$)	6(4,9)	8(6,11)	− 3.69^b^	< 0.001*
NT-pro BNP[pg/ml, M(Q_1_,Q_3_)]	48.8(25.4,104)	37.05(14.60,71.30)	2.35^b^	0.02*
CK-MB[U/L,M(Q_1_,Q_3_)]	15(13,19)	16(13,19)	− 1.37^b^	0.17
CRP[mg/L, M(Q_1_,Q_3_)]	0.90(0.40,2.15)	1.25(0.70,3.00)	− 2.30^b^	0.02*
D-dimer[mg/L, M(Q_1_,Q_3_)]	0.19(0.19,0.29)	0.19(0.19,0.25)	0.66^b^	0.51
ALT [U/L, M(Q_1_,Q_3_)]	18(14,26)	23(17,33)	− 2.75^b^	0.01*
AST [U/L, M(Q_1_,Q _3_)]	19(16,23)	19(16,24)	− 0.64^b^	0.52
GFR (ml/min/1.73m^2^,${\overline{\text{x}}} \pm {\text{s}}$	76.07 ± 17.57	78.24 ± 16.80	− 0.94	0.35
Creatinine[μmol/L, M(Q_1_,Q_3_)]	73(61,84)	74(62,85)	0.31^b^	0.76
Glucose[mmol/L,M(Q_1_,Q_3_)]	5.7 (5.1,7.3)	5.8(5.1,6.8)	0.06^b^	0.95
Glycated albumin(%,${\overline{\text{x}}} \pm {\text{s}}$)	14.66 ± 2.94	14.70 ± 3.68	− 0.11	0.91
HbA_1_c[%, M(Q_1_,Q_3_)]	5.7(5.4,6.1)	5.8(5.6,6.4)	− 2.20^b^	0.03*
TC (mmol/L, ${\overline{\text{x}}} \pm {\text{s}}$)	4.02 ± 1.11	4.08 ± 1.05	− 0.39	0.7
TG (mmol/L, ${\overline{\text{x}}} \pm {\text{s}}$)	1.71 ± 1.17	1.85 ± 1.07	− 0.96	0.34
HDL-c(mmol/L, ${\overline{\text{x}}} \pm {\text{s}}$)	1.22 ± 0.32	1.13 ± 0.28	2.41	0.02*
LDL-c(mmol/L, ${\overline{\text{x}}} \pm {\text{s}}$)	2.06 ± 0.86	2.18 ± 0.89	− 1.02	0.31
TSH (μIU/ml, ${\overline{\text{x}}} \pm {\text{s}}$)	2.16 ± 1.59	1.94 ± 1.42	1.09	0.27

a,${\mathrm{x}}^{2} \mathrm{value};$ b, z value; BMI, body mass index; BP, blood pressure; DM, diabetes mellitus; HBP, high blood pressure; hs-cTnT, high sensitivity cardiac troponin; NT-pro BNP, brain natriuretic peptid; CK-MB, isoenzyme of creatine kinase; CRP, C-reactive protein; ALT, alanine transaminase; AST, aspartate transaminase; GFR, glomerular filtration rate; HbA_1_C, glycated hemoglobin; TC, total cholesterol; TG, total triglycerides; HDL-c, high-density lipoprotein cholesterol; LDL-c, low-density lipoprotein cholesterol; TSH, thyrotropin-releasing hormone

* *p* <0.05

### Parameters in echocardiography between obesity and non-obesity

In comparison with the non-obesity group, the obesity group had higher echocardiography’s parameters, with atrioventricular enlargement and wall thickening as main manifestations. More specifically, obese individuals showed higher values of indicators representing cardiac hypertrophy such as LAD, LVEDD, LVEDS, IVST and PWT in Table 2. However, no significant difference in cardiac systolic or diastolic function was observed.

**Table 2:** Parameters in echocardiography in 383 patients

	BMI		t value	*p* value
	Non-obesity (n = 313)	Obesity (n = 70)		
AAO(mm, ${\overline{\text{x}}} \pm {\text{s}}$)	1.51 ± 0.04	1.52 ± 0.04	− 0.98	0.33
LAD(mm, ${\overline{\text{x}}} \pm {\text{s}}$)	37.48 ± 3.78	40.83 ± 3.49	− 6.58	< 0.001*
LVEDD(mm, ${\overline{\text{x}}} \pm {\text{s}}$)	45.80 ± 3.69	48.30 ± 4.04	− 4.87	< 0.001*
LVEDS(mm, ${\overline{\text{x}}} \pm {\text{s}}$)	29.22 ± 2.63	31.05 ± 3.09	− 4.91	< 0.001*
IVST(mm, ${\overline{\text{x}}} \pm {\text{s}}$)	9.53 ± 1.26	10.15 ± 1.06	− 3.71	< 0.001*
PWT(mm, ${\overline{\text{x}}} \pm {\text{s}}$)	9.08 ± 1.01	9.58 ± 0.88	− 3.64	< 0.001*
PASP(mmHg, ${\overline{\text{x}}} \pm {\text{s}}$)	31.55 ± 3.92	32.02 ± 3.04	− 0.90	0.37
DT(ms, ${\overline{\text{x}}} \pm {\text{s}}$)	181.75 ± 28.76	185.57 ± 31.83	− 0.95	0.34
LVEF(%, ${\overline{\text{x}}} \pm {\text{s}}$)	65.51 ± 3.38	64.85 ± 4.08	1.22	0.23

AAO, aortic root angiogram; LAD, left atrial dimension; LVEDD, left ventricular end-diastolic dimension; LVEDS, left ventricular end-systolic dimension; IVST, interventricular septal thickness; PWT, posterior wall thickness; PASP, pulmonary artery systolic pressure; DT, e peak deceleration time; LVEF, left ventricular ejection fraction

* *p* <0.05

### Independent correlation between BMI and circulating hs-cTnT

As shown in Table 3, the adjusted β(95%CI) for circulating hs-cTnT exhibited a proportional relationship with BMI in subgroup analysis based on the presence or absence of coronary heart disease (CHD). Multiple linear regression was adopted with the non-obese being defined as the reference. Both in non-CHD and CHD group, β(95%CI) values for circulating hs-cTnT exhibited a proportional relationship with BMI regardless of whether possible confounders were adjusted. In unadjusted model, β values in non-CHD and CHD respectively were 2.89 (95%CI, 1.43 to 4.36) and 5.64 (95%CI, 1.78 to 9.5). In order to ensure the credibility of the research results, we have adjusted the known confounding factors. When age and sex were adjusted, β values in non-CHD and CHD respectively stood at 2.80 (95%CI, 1.43 to 4.16) and 6.62 (95%CI, 2.63 to 10.62). Additional adjustment for DM, HBP, hyperlipidemia, systolic BP, diastolic BP, CK-MB, NT-pro BNP, CRP, D-dimmer, ALT, AST, GFR, creatinine, glucose, glycated albumin, HbA1c, TC, TG, HDL-c, LDL-c and TSH led to a slight attenuation of the β values both in non-CHD and CHD, respectively standing at 2.22(95%CI, 0.73 to 3.71) and 5.58(95%CI, 0.70 to 10.46).

**Table 3:** β value of hs-cTnT in the study group according to BMI

Degree of coronary stenosis	Model 1	Model 2	Model 3
	β(95%CI)	β (95%CI)	β(95%CI)
*[0, 50%)*			
non-obesity	Reference	Reference	Reference
Obesity	2.89(1.43,4.36)	2.80(1.43,4.16)	2.22(0.73,3.71)
*[50%,75%)*			
non-obesity	Reference	Reference	Reference
Obesity	5.64(1.78,9.50)	6.62(2.63,10.62)	5.58(0.70,10.46)

CI, confidential interval

Model 1: unadjusted

Model 2: adjusted for age and sex

Model 3: adjusted for age, sex, DM, HBP, hyperlipidemia, systolic BP, diastolic BP, CK-MB, NT-pro BNP, CRP, D-dimer, ALT, AST, GFR, creatinine, glucose, glycated albumin, HbA1c, TC, TG, HDL-c, LDL-c and TSH

### Spearman correlation analysis showed a significant relationship between BMI and cardiac remodeling

The association between BMI and parameters in echocardiography or NT-pro BNP is exhibited in Table 4. BMI has shown an apparently positive correlation with LAD, LVEDD, LVEDS, IVST, PWT and NT-pro-BNP (rho = 0.4985, 0.3723, 0.3698, 0.3852, 0.3533 and -0.17; all *p* < 0.001) in spearman correlation analysis, potentially suggesting that obesity may be accompanied by cell enlargement and hypertrophy. However, BMI had no association with ventricular systolic or diastolic function.

**Table 4:** Association between BMI and parameters in echocardiography or NT-pro BNP

	AAO	LAD	LVEDD	LVEDS	IVST	PWT	DT	PASP	LVEF	NTpro-BNP
Rho	0.1809	0.4985	0.3723	0.3698	0.3852	0.3533	0.0604	− 0.0492	− 0.0794	− 0.17
*p* value	< 0.001*	< 0.001*	< 0.001*	< 0.001*	< 0.001*	< 0.001*	0.2597	0.3540	0.1344	< 0.001*

* *p*<0.05

### Spearman correlation analysis showed a significant relationship between cardiac remodeling and circulating hs-cTnT

The Rho for the association between parameters in echocardiography or NT-pro BNP and circulating hs-cTnT was displayed in Table 5. There existed a significantly positive correlation between circulating hs-cTnT and LAD, LVEDD, LVEDS, IVST, PWT and NT-pro-BNP (rho = 0.2705, 0.1300, 0.1378, 0.2256, 0.1742 and 0.1049; all *p* < 0.05) in spearman correlation analysis, potentially indicating a significant relationship between cardiac hypertrophy and circulating hs-cTnT. However, circulating hs-cTnT had no association with cardiac systolic or diastolic function, which showed the subclinical myocardial injury caused by obesity is earlier than abnormal cardiac function.

**Table 5:** Association between parameters in echocardiography or NT-pro BNP and hs-cTnT

	AAO	LAD	LVEDD	LVEDS	IVST	PWT	DT	PASP	LVEF	NTpro-BNP
Rho	0.2469	0.2705	0.1300	0.1378	0.2256	0.1742	0.0109	0.0455	− 0.0297	0.1049
*p* value	< 0.001*	< 0.001*	< 0.05*	< 0.05*	< 0.001*	< 0.001*	0.8394	0.3913	0.5758	< 0.05*

* *p* <0.05

### Hs-cTnT differed between non-obesity and obesity groups and correlated with BMI

**Fig. 1:**
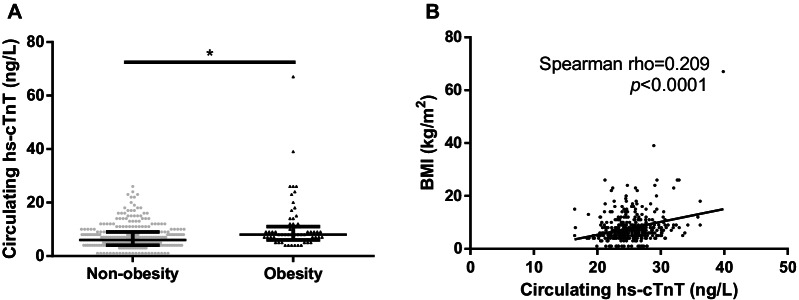
Hs-cTnT differs between groups and correlates with BMI. Circulating hs-cTnT levels in the two groups, shown in Median, IQR (**A**). Correlation between circulating hs-cTnT and BMI (**B**). *p < 0.05

The level of circulating hs-cTnT in obesity group was significantly higher than its counterpart in Fig. 1a. Meanwhile, BMI had shown a positive correlation with circulating hs-cTnT, with its rho standing at 0.209 (*p* < 0.001) in Fig. 1b.

## Discussion

The number of individuals with myocardial injury is steadily growing worldwide. Obese individuals are at high risk of myocardial injury than their non-obese counterparts. At the same time, considering that few studies involve the correlation between obesity and subclinical myocardial injury after excluding the impact of CHD, necessitating the emergence of our research. Briefly, our research had the following major findings on the premise of excluding the impact of other possible confounding factors: (i) There exists differences in circulating hs-cTnT, echocardiographic indexes and the inflammatory marker CRP between the obese group and the non-obese group, but no difference is displayed in the degree of coronary stenosis. (ii) Compared with the non-obese, BMI is independently associated with elevated but still within the normal range of circulating hs-cTnT independent of the degree of coronary stenosis; BMI is linked with early cardiac remodeling. (iii) Before cardiac diastolic and systolic function are affected, there is a certain correlation between slightly elevated circulating hs-cTnT and early cardiac remodeling.

Our findings are in an accordance with some community-based studies which explored the association between BMI and circulating hs-cTnT in continuous and categorical analyses. The ARIC (Atherosclerosis Risk in Communities) study [16] recruited 9,507 participants without baseline CAD to assess the association between BMI and circulating hs-cTnT in cross-sectional stage. In categorical analysis, the authors demonstrated that higher BMI groups were linked with a heightened probability of high circulating hs-cTnT levels (≥ 14 ng/L) even after all possible risk factors were adjusted. In continuous analysis, an independent, linear relationship between BMI and high circulating hs-cTnT levels were finalized when BMI was modeled continuously using a restricted cubic spline. In our study with all known confounders especially the presence of CHD being taken into account, we found that obesity remained independently related to subclinical myocardial injury. Of note, we conducted a stratified analysis of the degree of coronary stenosis and the correlation remained robust, thus providing confirmatory results for the study. In this regard, we believe that more attention should be given to non-ischemic subclinical myocardial injury related to obesity.

Indeed, the role of obesity in cardiovascular disease (CVD) is achieved through mechanisms such as inflammation, oxidative stress, fibrosis and alteration in cardiac function [17–21]. Meanwhile, numerous studies have revealed the potential role of adipose tissue (AT) inflammation to obesity-linked insulin resistance [22, 23]. Assessing the association between circulating hs-cTnT and CRP was beyond the present study’s reach. However, we speculated that the difference in systematic inflammation between the obese and the non-obese may be one of the pathological mechanisms leading to subclinical injury. Previous animal studies have suggested that obesity and obesity-linked insulin resistance threaten to paralyze mitochondrial function and fatty acids, causing elevated oxidative stress, release of inflammatory cytokines such as tumor necrosis-α (TNF-α) and interleukin-6 (IL-6) and an imbalance in adipokines [24–26]. These disorders are responsible for asymptomatic myocardial injury in obesity.

Persistent increase in troponin would further cause clinical cardiac remodeling [27]. We demonstrated that circulating hs-cTnT was associated with early cardiac remodeling before systolic and diastolic functions of the heart being not affected. Unanimously, Ravassa and Kuznetsova et al. [28] found that subclinical LV and LA remodeling was related to myocardial injury, with alternation in circulating hs-cTnT being the main manifestation. Additionally, our study also found that BMI is associated with early cardiac remodeling. Admittedly, cardiac remodeling is deemed as a preclinical form of HF that predisposes to subsequent HF. An animal model [29, 30] using cTnT as an inducer of immune myocarditis could be sufficient to explain why obese individuals with a seemingly healthy heart could progress into overt HF. The continuous presence of circulating hs-cTnT induces the sustained release of cardiomyocytes’ local inflammation, followed by cardiomegaly, fibrosis, reduced cardiac functions and 30% mortality over 270 days. In published population-based studies [31–33], the correlation between obesity and late cardiac remodeling or even HF has been verified and reached a conclusion. A cross-sectional study based in South Korea found that the increased BMI was closely associated with both structural and functional left ventricular (LV) abnormalities [34]. The Framingham Heart Study showed that obese subjects had a doubling of the risk of HF compared with subjects with a normal BMI after a 14-year-long follow-up [32].

Circulating cardiac troponin levels are routinely used as a diagnostic tool for ACS but also served as differentiating myocardial injury from CHD [8]. Certainly, the efficacy of circulating cardiac troponin as forecasters of cardiovascular events in non-ischemic heart disease has been testified. A mild increase in circulating cardiac troponin has been a sign of myocardial injury and is associated with future CHD events, which was testified by a recent study. [9, 10] Thus, assessment of myocardial injury among obese individuals appears to be significantly important since a mild increase in circulating cardiac troponin within the normal range has shown a relationship with future cardiovascular events. We concentrated on circulating hs-cTnT to detect subclinical myocardial injury when exploring the association between obesity and circulating hs-cTnT. Thus, findings from our study indicate that it is necessary for obese individuals to carry out regular screening of myocardial injury markers to avoid potentially subsequent cardiovascular events.

The innovation of this study lies in the fact that we finalized that obesity is related to slightly elevated troponin independent of CHD, implying a brand-new viewpoint that non-ischemic myocardial injury is accompanied by obesity although people has not evolved into HF. Most importantly, our discovery makes the chain of HF’s development and occurrence appear to be more complete, potentially deepening people’s understanding of HF.

## Conclusions

To sum up, our study has confirmed (that) the contribution of obesity to the slightly elevated circulating hs-cTnT when CHD is accounted for, which potentially indicates the independent impact of obesity on non-ischemic subclinical myocardial injury. And efforts to promote optimal body weight may reduce the risk of non-ischemic subclinical myocardial injury. Management of risk factors for subclinical myocardial injury and preclinical phases of HF should be prioritized considering the alarming trend toward growing obese population globally.

## Limitations

Our study serving as a cross-sectional study was potentially flawed with selection bias and risks for confounding factors. Secondly, for a lack of further follow-up, we failed to detect the progression of individuals either obese or not for further obtaining a stronger evidence for the causal relationship between obesity and myocardial injury, cardiac remodeling or even impaired cardiac function. Third, the relatively small sample size may threaten to testify the validity of our findings.

## Data Availability

The datasets used and analyzed during the current study are available from the corresponding author on reasonable request.
